# Antagonistic and plant growth promotion of rhizobacteria against *Phytophthora colocasiae* in taro

**DOI:** 10.3389/fpls.2022.1035549

**Published:** 2022-12-02

**Authors:** Bekele Gelena Kelbessa, Farideh Ghadamgahi, P. Lava Kumar, Rodomiro Ortiz, Stephen C. Whisson, Ranjana Bhattacharjee, Ramesh Raju Vetukuri

**Affiliations:** ^1^ Department of Plant Breeding, Swedish University of Agricultural Sciences, Lomma, Sweden; ^2^ International Institute of Tropical Agriculture, Ibadan, Nigeria; ^3^ Cell and Molecular Sciences, James Hutton Institute, Dundee, United Kingdom

**Keywords:** *Colocasia esculenta*, *Phytophthora colocasiae*, biocontrol, growth promotion, inhibitory effect, plant growth-promoting bacteria

## Abstract

Taro leaf blight caused by *Phytophthora colocasiae* adversely affects the growth and yield of taro. The management of this disease depends heavily on synthetic fungicides. These compounds, however, pose potential hazards to human health and the environment. The present study aimed to investigate an alternative approach for plant growth promotion and disease control by evaluating seven different bacterial strains (viz., *Serratia plymuthica*, S412; *S. plymuthica*, S414; *S. plymuthica*, AS13; *S. proteamaculans*, S4; *S. rubidaea*, EV23; *S. rubidaea*, AV10; *Pseudomonas fluorescens*, SLU-99) and their different combinations as consortia against *P. colocasiae*. Antagonistic tests were performed in *in vitro* plate assays and the effective strains were selected for detached leaf assays and greenhouse trials. Plant growth-promoting and disease prevention traits of selected bacterial strains were also investigated *in vitro*. Our results indicated that some of these strains used singly (AV10, AS13, S4, and S414) and in combinations (S4+S414, AS13+AV10) reduced the growth of *P. colocasiae* (30−50%) *in vitro* and showed disease reduction ability when used singly or in combinations as consortia in greenhouse trials (88.75−99.37%). The disease-suppressing ability of these strains may be related to the production of enzymes such as chitinase, protease, cellulase, and amylase. Furthermore, all strains tested possessed plant growth-promoting traits such as indole-3-acetic acid production, siderophore formation, and phosphate solubilization. Overall, the present study revealed that bacterial strains significantly suppressed *P. colocasiae* disease development using *in vitro*, detached leaf, and greenhouse assays. Therefore, these bacterial strains can be used as an alternative strategy to minimize the use of synthetic fungicides and fertilizers to control taro blight and improve sustainable taro production.

## 1 Introduction

Taro (*Colocasia esculenta*) is a tropical plant of Asian origin that is widely cultivated in different parts of the world, including Africa, Oceania, Asia, and South America (Huang et al., 2007; Vetukuri et al., 2018; Ahmed et al., 2020; Bellinger et al., 2020). Globally, taro is the fifth most cultivated monocotyledonous root crop in the *Araceae* family, and its production is dominated by Cameroon, Nigeria, Ghana, Ethiopia, and China, which account for 81.9% of total taro production worldwide (Grimaldi and van Andel, 2018; Mitharwal et al., 2022; Oladimeji et al., 2022). The crop is mainly cultivated for its starchy corm (underground stem), which is the main source of carbohydrates as an energy source and a staple food in tropical and semi-tropical regions of the world (Matthews, 2004; Liu et al., 2006; Kaushal et al., 2015). In addition to corms, the leaves of the crop are also used as a vegetable, as they are rich in protein, dietary fiber, micronutrients, and vitamin C, depending on the cultivar (He et al., 2013; Gupta et al., 2019; Mitharwal et al., 2022). Both corms and leaves of taro crops are rich in health-promoting substances that may protect against a variety of human diseases such as tuberculosis, fungal infections, and pulmonary congestion (Pereira et al., 2018; Pachiappan et al., 2020).

Although the crop has immense potential to contribute to global food security, its production is often limited by various biotic and abiotic stresses (Sahoo et al., 2007; Bi et al., 2020). Among biotic stresses, taro leaf blight (TLB) caused by *Phytophthora colocasiae*, a hemibiotrophic oomycete, is one of the most devastating diseases of taro plants worldwide (Adomako et al., 2017). The pathogen attacks leaves and petioles and leads to a reduction in photosynthesis, which in turn reduces corm yield by more than 50% in susceptible cultivars (Misra et al., 2008; Feng et al., 2022). In addition to reducing yield in an infected plant, corm rot can also occur, leading to severe yield losses in storage (Devi et al., 2016). The pathogen has spread through the planting of infected corms, and splash and waterborne sporangia and zoospores, threatening food security and farmer welfare in taro-growing regions (Lebot et al., 2003; Singh et al., 2012; Feng et al., 2022). The devastating effects of the disease continues to affect the livelihoods of subsistence farmers and rural communities in humid tropical and semi-tropical areas of the world (Singh et al., 2012; Feng et al., 2022). Several strategies are used to control taro leaf blight, including cultural practices, synthetic chemicals, and host resistance. Although some breeding efforts have been made to develop TLB-resistant cultivars (Brooks, 2008; Bellinger et al., 2020), the popularization of these resistant cultivars is limited due to the lack of other desirable economic and market traits of the plant (Nath et al., 2014; Oladimeji et al., 2022). Despite the efficacy of chemical fungicides, control of TLB disease is not economically viable due to the waxy coating of leaf laminae and the emergence of fungicide-resistant strains (Nath et al., 2013). Moreover, the application of fungicides poses a number of problems, including potential hazards to human health, alteration of beneficial microbes associated with plants, environmental pollution, and cost to growers (Nath et al., 2015; Bellinger et al., 2020; Wang et al., 2020; Zhang et al., 2021). Finding a cost-effective, sustainable, and environmentally friendly strategy to control this disease and maintain sustainable production of this crop is therefore urgent.

The use of plant growth-promoting rhizobacteria (PGPR) inhabiting plants as biocontrol agents and bio-fertilizers to improve plant health and growth under both normal and adverse environmental conditions are becoming popular as a sustainable strategy to control plant diseases (Morrison et al., 2017; Ab Rahman et al., 2018). PGPR can directly improve plant growth by synthesizing growth-stimulating hormones, fixing nitrogen, dissolving phosphates, and supporting iron supply (Moto et al., 2017; Köhl et al., 2019; Rodriguez et al., 2020). PGPR also indirectly improves the health status of its host by producing antimicrobial compounds and hydrolytic enzymes that inhibit the proliferation of pathogens. Species of the bacterial genus *Serratia* and *Pseudomonas* have been reported to have biocontrol activity against a variety of plant pathogens, but none have been tested for their ability to inhibit taro leaf blight. The aim of the present study was to investigate the biocontrol efficacy and plant growth promotion potential of seven bacterial strains and compatible combinations as consortia against *P. colocasiae*.

## 2 Materials and methods

### 2.1 Chemical and culture media

Luria Bertani (LB) broth (Sigma-Aldrich, Taufkirchen, Germany) was prepared by dissolving 25 g L^-1^ in sterilized distilled H_2_O, autoclaving, and used for culturing bacterial strains. Vietnamese strain 7290 of *P. colocasiae* was grown on V8-media [CaCO_3_, 1.5 g L^-1^; V8 vegetable juice, 100 g L^-1^; and agar, 15 g L^-1^) (Vetukuri et al., 2018). Peptone-agar [peptone, 10 g L^-1^; NaCl, 5 g L^-1^; CaCl_2_.2H_2_O, 0.1 g L^-1^; agar, 16 g L^-1^] was prepared and used for the lipase activity assay. Tryptic Soy Agar (TSA) (Sigma-Aldrich, Taufkirchen, Germany) was used for compatibility, amylase, and hydrogen cyanide assays.

### 2.2 Antagonism assay

Seven bacterial strains were assessed in this study. Three of them (viz., *Serratia rubidaea*, EV23; *S. rubidaea*, AV10; and *Pseudomonas fluorescens*, SLU-99) were from our in-house collections and were originally collected from potato and tomato rhizosphere soil samples. The four other strains (viz., *Serratia plymuthica* S412 and; *S. plymuthica* S414 (Akhter, 2014) plus *S. plymuthica* AS13, and *S. proteamaculans* S4 (Gkarmiri et al., 2015) are known to suppress other plant pathogens, but antagonistic activity against *P. colocasiae* has not been determined. Hence, antagonistic activities of these individual strains against *P. colocasiae* were first evaluated. Subsequently, compatibility between the strains was studied, according to the method described by Santiago et al. (2017), to determine consortia that may have synergistic effects. Briefly, the strains were cultured overnight in LB broth at 28°C and shaking at 220 rpm. After measuring the optical density of each strain and setting the OD_600_ = 0.3, one strain was streaked in a straight line onto freshly prepared TSA plates and incubated at 28°C for 24 h. Afterward, the second strain was inoculated at a 90° angle starting from the colony streak of the first strain and incubated at 28°C for 94 h. After the incubation period, the zone of inhibition at the junction of the paired bacteria antagonist was assessed and photographed.

The antagonistic activities of the compatible strains along with all the single strains were subsequently tested in a dual culture confrontation assay *in vitro* against *P. colocasiae*. For this purpose, a sterile cork borer (4 mm diameter) was used to take a mycelial disk of *P. colocasiae* from the edge of an actively growing colony on V8 agar (2-weeks-old plates) and inoculated it into the center of a 90 mm diameter Petri dish containing freshly prepared V8 agar medium. Bacterial strains were cultured overnight in LB broth at 28°C and shaken at 220 rpm, and optical density at 600_nm_ was adjusted (OD_600_ = 0.3 for single strains and OD_600_ = 0.15 for combined strains). The strains were streaked in a straight line on both sides of Petri plates, 2 cm from the center, simultenously with pathogen inoculation. Plates were incubated at 28°C for 7 days and the zone of mycelial growth inhibition was calculated as described in (Wang et al., 2019). V8 medium (with only *P. colocasiae*) was used as a negative control. The assay was done with six replicates per treatment and repeated twice. The zone of inhibition (ZI) was estimated as follows:


$$ZI\;\left(\%\right)=\frac{\left(R1-R2\right)}{R1}x\;100$$

where R1 was the diameter of mycelia growth in untreated control and R2 was the diameter of mycelia growth in the presence of bacterial antagonists.

### 2.3 Assay of antimicrobial activity of cell-free supernatant

To evaluate the antimicrobial activity of cell-free culture supernatants of selected strains against *P. colocasiae*, each strain was cultured overnight in LB broth at 28°C and the optical density was adjusted (OD_600_ = 0.2 for single strain and 0.1 for combined strains) and grown overnight at 28°C with shaking (220 rpm). Afterward, bacterial cultures were centrifuged (4200 × g for 10 min, 4°C) to collect the supernatant, which was then filtered using a sterile Filtropur S 0.2 µm (Sigma Aldrich, Germany). The cell-free supernatant was then immediately added to pre-cooled Corn Meal Agar (CMA) at a concentration of 10% (v/v) according to the method described by Ali Abdulameer et al. (2017) with some modifications, and 15 ml of the prepared CMA medium was poured into 60 mm diameter Petri dishes. Then, a 1 cm mycelial plug of a 14-day-old culture of *P. colocasiae* was inoculated into the centre of each Petri dish. Corn Meal Agar amended with LB medium served as the control. The assay was performed in triplicate and incubated at 28°C for 10 days. After 10 days of incubation, radial growth inhibition of *P. colocasiae* was measured soon after the growth of the pathogen reached 60 mm diameter in the control plates, and the percentage of inhibition was recorded.

### 2.4 Disease suppression and plant growth-promotion traits

#### 2.4.1 Biochemical and enzyme activity assays

##### 2.4.1.1 Biofilm production test

Biofilm production by selected bacterial strains was assessed using a microplate assay method (Pratt and Kolter, 1998). Ten µL of either a single strain (OD_600_ = 0.3) or combined strains (OD_600_ = 0.15) were transferred to 96-well polystyrene microplates filled with 150 µL of LB broth and incubated at 28°C for 48 h. To detect biofilm production, unbound bacteria were removed from the wells, rinsed three times with sterilized distilled H_2_O (dH_2_O), air dried, and then stained with 0.2% crystal violet (170 µL). After 30 min of incubation at room temperature, the crystal violet was discarded, and the wells were again washed three times with sterilized dH_2_O and de-stained with 96% ethanol (200 µL). Subsequently, 100 µL from each well was transferred to a new microtiter plate and absorbance was measured at 595_nm_ using a spectrophotometer (Thermo Fisher Scientific, Ratastie 2*, *Fi*-*01620 Vantaa*, *Finland). The biofilm assay was performed twice, with eightfold technical replication per strain, and sterile LB media was used as a control. The results obtained were adjusted for background staining by statistically subtracting the value for crystal violet bound to the untreated controls from the treated samples.

#### 2.4.2 Lytic enzyme production

##### 2.4.2.1 Protease activity

The ability of the selected bacterial antagonists to produce proteases was evaluated on skim milk (SM) agar [skim milk, 28 g L^-1^; dextrose, 1 g L^-1^; casein hydrolysate, 5 g L^-1^; yeast extract, 2 g L^-1^; agar, 15 g L^-1^] at pH 7 ± 0.2 according to the published method (Caulier et al., 2018). Ten µL of either single (OD_600_ = 0.3) or combined strains (OD_600_ = 0.15) were pipetted onto the above medium and incubated at 28°C for 48 h. After the incubation period, the appearance of a clear zone around the bacterial colony was assessed. This assay was conducted with three technical replicates per strain and repeated twice.

##### 2.4.2.2 Amylase activity

Amylase production of selected strains was studied on TSA plates (TSA, 40 g L^-1^, and soluble starch, 2 g L^-1^) according to the method described by Pascon et al. (2011) with some modifications. Ten µL of either single or combined strains were spotted onto TSA plates with soluble starch and incubated at 28°C for 48 h. The plates were then flooded with an iodine solution (1% potassium iodide and 0.1% iodine) for 5 min, and amylase activity was assessed by the starch degradation zone formed on the purple background (Pascon et al., 2011). The experiment was done with three replicates per strain and repeated twice.

##### 2.4.2.3 Cellulase activity

Cellulase-producing strains were assayed on carboxymethyl cellulose (CMC) agar [KH_2_PO_4_, 1 g L^-1^; MgSO_4_.7H_2_O, 0.5 g L^-1^; NaCl, 0.5 g L^-1^; FeSO_4_.7H_2_O, 0.01 g L^-1^; NH_4_NO_3_, 0.3 g L^-1^; CMC, 10 g L^-1^; agar, 12 g L^-1^] at pH 7 ± 0.2 according to published methods (Ariffin et al., 2006; Caulier et al., 2018). About 10 µL of the single or combined bacterial antagonists were spotted onto CMC agar and incubated at 28°C for 120 h. Accordingly, the cellulase-degrading ability of the strains was confirmed by superfusing the plates with 0.1% Congo red for 15 min followed by 1M NaCl (Thomas et al., 2018). The cellulase assay was performed with six technical replicates and repeated twice.

##### 2.4.2.4 Lipase activity

Qualitative screening was performed to evaluate the lipase-producing abilities of the strains in peptone agar medium following the published method (Haq et al., 2020) with some modifications: the assay was conducted at pH 6 and supplemented with 1% separately sterilized Tween 20. Ten µL of either single or combined strains were pipetted onto peptone agar medium and incubated at 28°C for 120 h. A clear zone formed around a colony was used as an indicator of the lipase activity of the bacterial strains. The lipase activity assay was done with six replicates and repeated twice.

##### 2.4.2.5 Hydrogen cyanide

For qualitative screening of hydrogen cyanide production by selected bacterial strains, the previous published method was used with some modification (Reetha et al., 2014). Bacterial strains were cultured overnight in LB medium and 10 µL of either single or combined selected strains were spread on TSA plates supplemented with 4.4 g L^-1^ glycine. The top of the Petri plate was covered with sterilized filter paper saturated with 0.5% picric acid (2,4,6-trinitrophenol) and 2% Na_2_CO_3_. The plates were then sealed and incubated at 28°C for 48 h. The intensity of the color change of filter paper from yellow to reddish brown was recorded as a positive reaction of cyanogenic activity. *Pseudomonas chlororaphis*, FG294, which is known to produce hydrogen cyanide, was used as a positive control. The test for hydrogen cyanide production was carried out with six replicates per strain or combination, and repeated twice.

##### 2.4.2.6 Chitinase

For qualitative evaluation of the chitinase activity of the strains, colloidal chitin was prepared according to the method previously described by Roberts and Selitrennikoff (1988). Afterwards, selected strains were cultured overnight, and 10 µL of either single or combined strains were spotted onto colloidal chitin (CC) agar [NH_4_H_2_PO_4_, 1 g L^-1^; KCl, 0.2 g L^-1^; MgSO_4_.7H_2_O, 0.2 g L^-1^; CC, 10 g L^-1^, and agar, 20 g L^-1^] at pH 6 ± 0.2, according to published method (Caulier et al., 2018) and incubated at 28°C for 120 h with three technical replicates per treatment.

### 2.5 Plant growth-promoting traits

#### 2.5.1 Siderophore production assay

The potential of bacterial strains to secrete siderophores was tested on modified chromazurol S (CAS) agar [10 mL FeCl_3_.6H_2_O; 27 mg/100 mL HCl 10 mM; 50 mL CAS, 1.2 g L^-1^; 40 mL hexadecyltrimethylammonium bromide (HDTMA), 1.82 g L^-1^; 900 ml king broth agar] at a pH of 6.8 ± 0.2 according to published method (Neilands, 1981; Louden et al., 2011). A 10 µL drop of either single (OD_600_ = 0.3) or combined strains (OD_600_ = 0.15) of overnight bacterial culture in LB medium was pipetted onto CAS agar and incubated at 28°C for 72 h with six replicates. Formation of an orange halo zone was indicative of siderophore production. The concentration of siderophores produced by the strains was estimated according to published method (Devi et al., 2016). In brief, bacterial strains were cultured overnight in LB medium at 28°C and then centrifuged at 4200 × g for 10 min. One mL of the supernatant was mixed with 1 mL of CAS reagent [10 mM HDTMA, 1 mM FeCl_3_, and 2 mM CAS solution]. The absorbance was then measured at 630_nm_, compared to the untreated control (1 mL untreated LB broth + 1 mL CAS reagent). The amount of siderophores in all samples was measured in siderophore units (SU, %) following the previously published method (Arora and Verma, 2017) as noted below:


$$SU\;\left(\%\right)\;=\frac{\left(Ar-As\right)}{Ar}x\;100$$

where Ar was the absorbance of untreated control and As the absorbance of strains at 630_nm_ (bacterial supernatant + CAS reagent).

#### 2.5.2 Auxin production assay (IAA)

The ability of the strains to produce IAA was examined following the published method (Martinez et al., 2018). Strains were cultured in LB broth containing 100 µg mL^-1^ or without L-tryptophan and incubated at 28°C for 48 h. After the incubation period, the strain suspension was centrifuged at 4200 × g for 10 min. One mL of supernatant was then transferred into 96-well white-bottom plates containing 200 µL of Salkowski reagent [15 mL 95-97% H_2_SO_4_, 0.75 mL 0.5 M FeCl_3_.6H_2_O, and 25 mL dH_2_0]. Absorbance at 530_nm_ was then measured using spectrophotometry (Thermo Fisher Scientific, Ratastie 2*, *Fi*-*01620 Vantaa*, *Finland) to determine the amount of IAA production by the strains. Sterile LB medium (with or without L-tryptophan) was used as a control. The IAA concentration at 530_nm_ of the control was subtracted from the concentration of indole-related compound at 530_nm_ of the bacterial strains to obtain the background subtraction concentration.

#### 2.5.3 Ammonia production

To detect ammonia production, all selected strains were grown in 4% peptone water broth and incubated for 48 h at 28°C. After the incubation period, 0.5 mL of Nessler reagent was added to the strain suspension and the development of a brown to yellow color indicated that the strains were able to produce ammonia (Bhattacharyya et al., 2020).

#### 2.5.4 Phosphorus solubilization

To determine the phosphorus solubilizing ability of the strains, an *in vitro* assay was carried out following the method described by Mehta and Nautiyal (2001). For this purpose, the selected strains were inoculated on a National Botanical Research Institute Phosphate (NBRIP) agar medium [glucose, 10 g L^-1^; Ca_3_(PO_4_)_2_, 0.5 g L^-1^; MgCl_2_, 0.5 g L^-1^; (NH_4_)_2_SO_4_, 0.1 g L^-1^; MgSO_4_. 7H_2_O, 0.25 g L^-1^; KCl, 0.2 g L^-1^; agar, 15 g L^-1^] and incubated at 28°C for 72 h. The formation of a distinct halo zone surrounding the spot inoculated bacteria colony after incubation is considered a positive reaction. From this assay the phosphate solubility index (PSI) of each tested strain was calculated (Marra et al., 2012) as follows:


$$PSI=\frac{Diameter\;of\;halo\;zone\;\left(mm\right)+Diameter\;of\;colony\;\left(mm\right)}{Diameter\;of\;colony\;\left(mm\right)}$$

### 2.6 Detached leaf assay

To evaluate the biocontrol potential of the strains *in planta* against *P. colocasiae*, a detached leaf assay was conducted with selected strains that showed better antagonistic effects in the *in vitro* dual culture assay. Taro corms of a susceptible cultivar were planted in 2 L plastic pots with sterilized compost (Krukvåxtjord Lera and Kisel, Sweden) and grown in the greenhouse at 20°C and under 72% relative humidity (RH) for four weeks. To minimize possible effects of leaf age in evaluating *P. colocasiae* infection, fully developed leaves of the same age were collected from four-week-old plants and rinsed with sterilized dH_2_O. Bacterial strains were grown overnight in LB broth at 28°C and the OD_600_ was adjusted to 0.3 for single strains and 0.15 for combined strains. The washed leaves were placed in plastic boxes and then were spot-inoculated (20 µL) with the bacterial strains one day before pathogen inoculation. The next day, 2 mL of sterile dH_2_O was poured onto 2-week-old plates of *P. colocasiae*, rubbed with a sterile spreader to dislodge sporangia and the supernatant was filtered through a sterile 40 µm cell strainer (Starlab, Germany). Afterward, the spore density of *P. colocasiae* was calculated using a 0.2 mm haemacytometer (Fuchs-Rosenthal, Germany) and the suspension was standardized to obtain a final sporangia concentration of 2 × 10^4^ spores/mL for the pathogen, of which 10 µL was spot-inoculated at the same site on the abaxial side of taro leaves inoculated with bacteria. The spot inoculated leaves were then incubated at 22°C and 72% RH for 120 h. After the incubation period, the inoculation sites were excised with a cork borer (1.8 cm diameter) and incubated in trypan blue solution for 30 min (Koch and Slusarenko, 1990), followed by overnight incubation in absolute ethanol (99.7%) at room temperature (Fernández-Bautista et al., 2016). Ethanol de-stained leaves were kept in a 30% glycerol solution for 30 min and images were then taken using an Epson Perfection V750 Pro scanner (J221A 24V 1.4A, Indonesia). Afterward, the infected area of the necrotic leaves was quantified using the NIH software ImageJ. Four treatments were included in this assay: (1) leaf + sterile dH_2_O; (2) leaf + pathogen; (3) leaf + single strain + pathogen; and (4) leaf + combined strains + pathogen. The experiment was conducted with seven technical replicates and repeated three times.

### 2.7 Greenhouse trials

A greenhouse experiment was conducted to evaluate the biocontrol and plant growth-promoting potential of the selected strains. Since the DLAs were preliminary results, the four strains showing the lowest mean disease lesion area, and their combinations (S414, S4, AS13, AV10, S4 + S414, and AS13 + AV10) were selected to further examine their biocontrol efficacy under realistic conditions (on the whole plant), although some other combined strains showed better suppression of the necrotic leaf lesion. For this purpose, bacterial strains were cultured overnight and centrifuged at 4200 × g for 20 min to collect the cells. The OD_600_ of the collected bacterial cells was adjusted to 0.3 for single strains and 0.15 for combined strains) in 1× phosphate buffered saline (PBS) and used to soak the taro corms for 30 min. The control treatment was soaked with 1x PBS only. Bacteria-soaked taro corms were then dried at room temperature for 30 minutes and planted in 1.5 L pots with sterilised compost and grown in the greenhouse at 20°C and 70% RH for 30 days. Since the pathogen attacks both the corms and leaves, the same strains used to treat the corms were sprayed on leaves (20 ml/pot, to cover entire foliage of the plant grown in a given pot) one day before spot inoculation of *P. colocasiae*. The leaves were then spot inoculated with the same final sporangia suspension concentration used in the DLAs, and the plants were kept at 20°C and 85% RH for five days. The daily development of disease symptoms was observed with each treatment and compared with the untreated control. Disease assessment was done by measuring the infected area of the leaves using a 0-4 rating scale of [0 = no visible disease symptoms; 1 = 1−25% (low infection); 2 = 26−50% (moderate infection); 3 = 51−75% (high infection); 4 = 76−100% (very high infection)] as described by Adinde et al. (2016). The mean leaf disease score was calculated as the disease severity index (DSI) (Chiang et al., 2017), was calculated for each plant by summing the single score for all taro plants as follows:


$$DSI\;\left(\%\right)=\frac{\left[sum\;\left(class\;frequency\;x\;score\;of\;rating\;class\right)\right]}{[\left(total\;number\;of\;observations\right)x\;\left(maximal\;disease\;index\right)}x100$$

The plant growth-promoting effect of four single strains (viz., S414, S4, AS13, and AV10) and two combinations (viz., S4 + S414, and AS13 + AV10) were also investigated separately in the greenhouse. For this purpose, taro corms were pre-soaked with the bacterial strains as mentioned above and grown for 30 days at 20°C and 70% RH, along with untreated control plants. At 14 days after planting, the same strains used for corm treatment (20 ml/pot) were carefully poured around the roots of the plants. Various growth parameters such as plant height, chlorophyll content, the whole plant fresh weight, dry weight, root weight, and corm weight were measured 30 days after treatment. This experiment was conducted with three treatments: (a) plant + sterile dH_2_O; (b) plant + single strain, and (c) plant + combined strains. Both the biocontrol and plant growth promotion trials were conducted with seven biological replicates per treatment in a completely randomized experimental design.

### 2.8 Statistical analysis

The initially obtained data for antagonism, biofilm, detached leaf, and greenhouse were subjected to the Shapiro-Wilk´s and Levene´s test (*P > 0.05*) to confirm the normal distribution of the data and homogeneous variance, respectively. Subsequently, the test data were statistically analyzed with the software R x 64 4.2.0 using Duncan’s Multiple Ranges at *P ≤ 0.05*, and the data were reported as mean values ± standard deviation.

## 3 Results

### 3.1 *In vitro* antagonism assay

**Figure 1:**
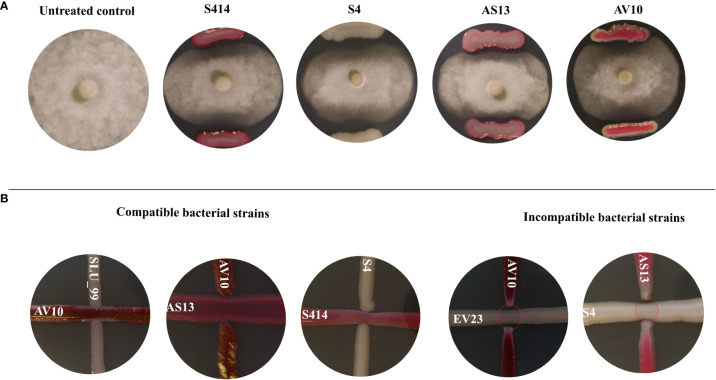
**(A)** Inhibition of *Phytophthora colocasiae* mycelia growth in dual culture assay. Control culture of *P. colocasiae* exhibited uniform hyphal growth, with the formation of abundant aerial hyphae and sporangiophores. Co-culture with biocontrol bacteria led to inhibition of radial hyphal growth and reduced formation of aerial structures; **(B)** Compatibility assay between co-inoculated strains. The strains were streaked at 90 degrees and their synergistic effect and zone of inhibition were evaluated after 96 h of incubation. Compatible strains showed no inhibition of each other and colonies merged. Incompatible strains showed clear zones of inhibition between the colonies (red ovals).

**Figure 2:**
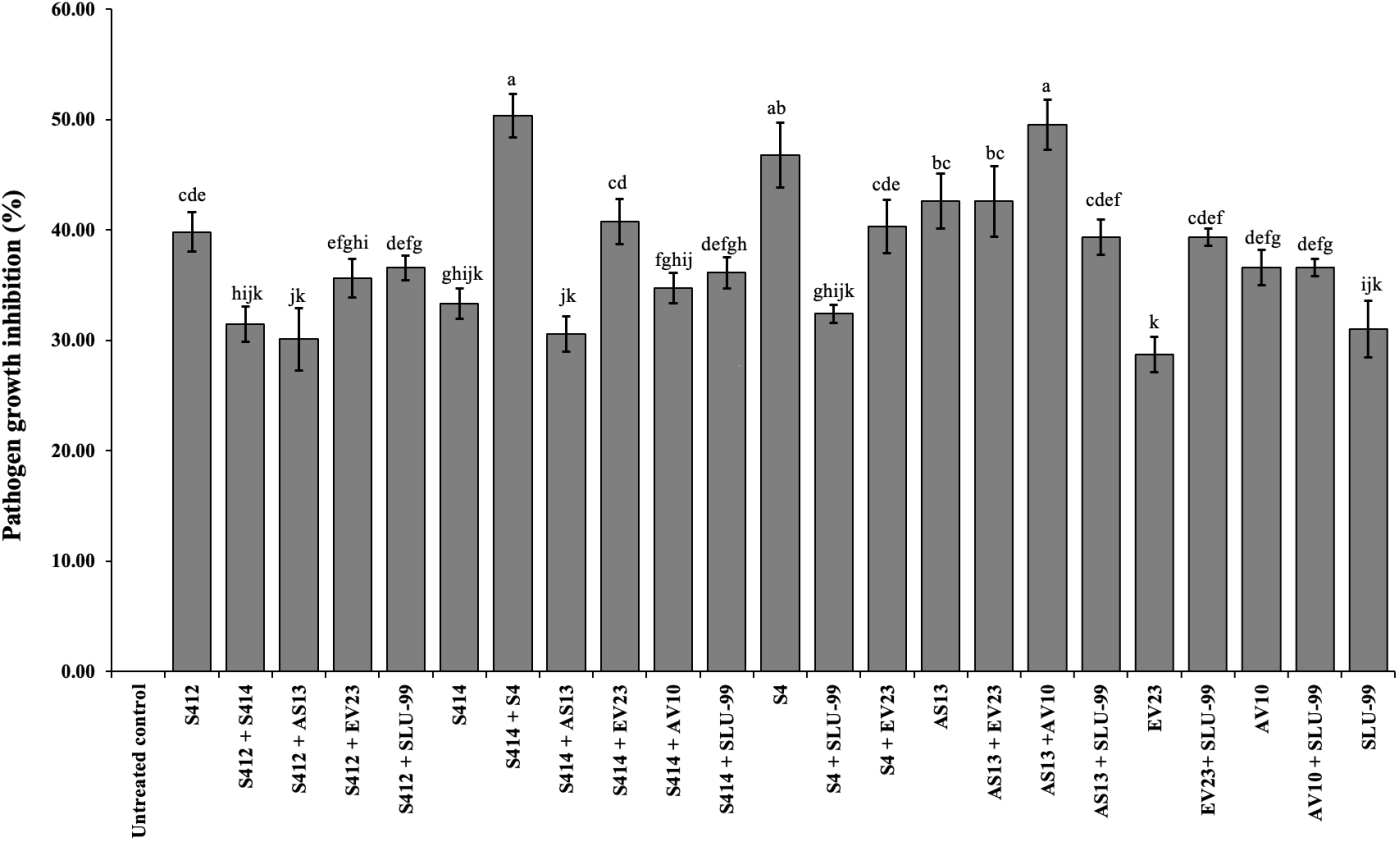
In vitro antagonistic effect of bacterial strains individually or in combination against mycelial growth of *Phytophthora colocasiae*. Data are the mean ± standard deviation (SD) of six technical replicates. Mean values with different letters indicate differences between tested strains according to Duncan’s multiple range test at *P* > 0.05.

Our results show that of 21 possible strain combinations, five strain combinations inhibited each other on TSA plates, regardless of the direction of spread (**Figure 1B**). In contrast, sixteen combinations were compatible and could grow over each other (**Figure 1B** and **Table 1**). The results of the *in vitro* antagonistic assay demostrated that *P. colocasiae* growth was suppressed by at least 30% when challenged with single or combined strains, but the efficiency of the strains was different (**Figures 1**, **2**). The highest growth reduction (49−50%) of the pathogen was observed in the presence of combined strains as consortia, followed by S4, where mycelial growth was reduced by 46.76% compared to the untreated control (**Figure 2**). In this study, we also investigated the antimicrobial activities of the cell-free supernatants of the strains against *P. colocasiae*. As shown in **Supplementary Figure S1**, the cell-free filtrate of the strains significantly reduced the radial growth of *P. colocasiae* compared with the untreated control. However, there was no significant difference between the tested single and combined strains in terms of their antimicrobial activities against the growth of the pathogen.

**Table 1:** Compatibility assay between co-inoculated strains.

Strains	S414	S4	AS13	EV23	AV10	SLU_99
S412	+	–	+	+	–	+
S414		+	+	+	+	+
S4			–	–	–	+
AS13				+	+	+
EV23					+	+
AV10						+

+ represents compatibility between co-inoculated strains, while – negative result represents incompatibility between co-inoculated strains.

### 3.2 Biofilm and enzymatic production

**Figure 3:**
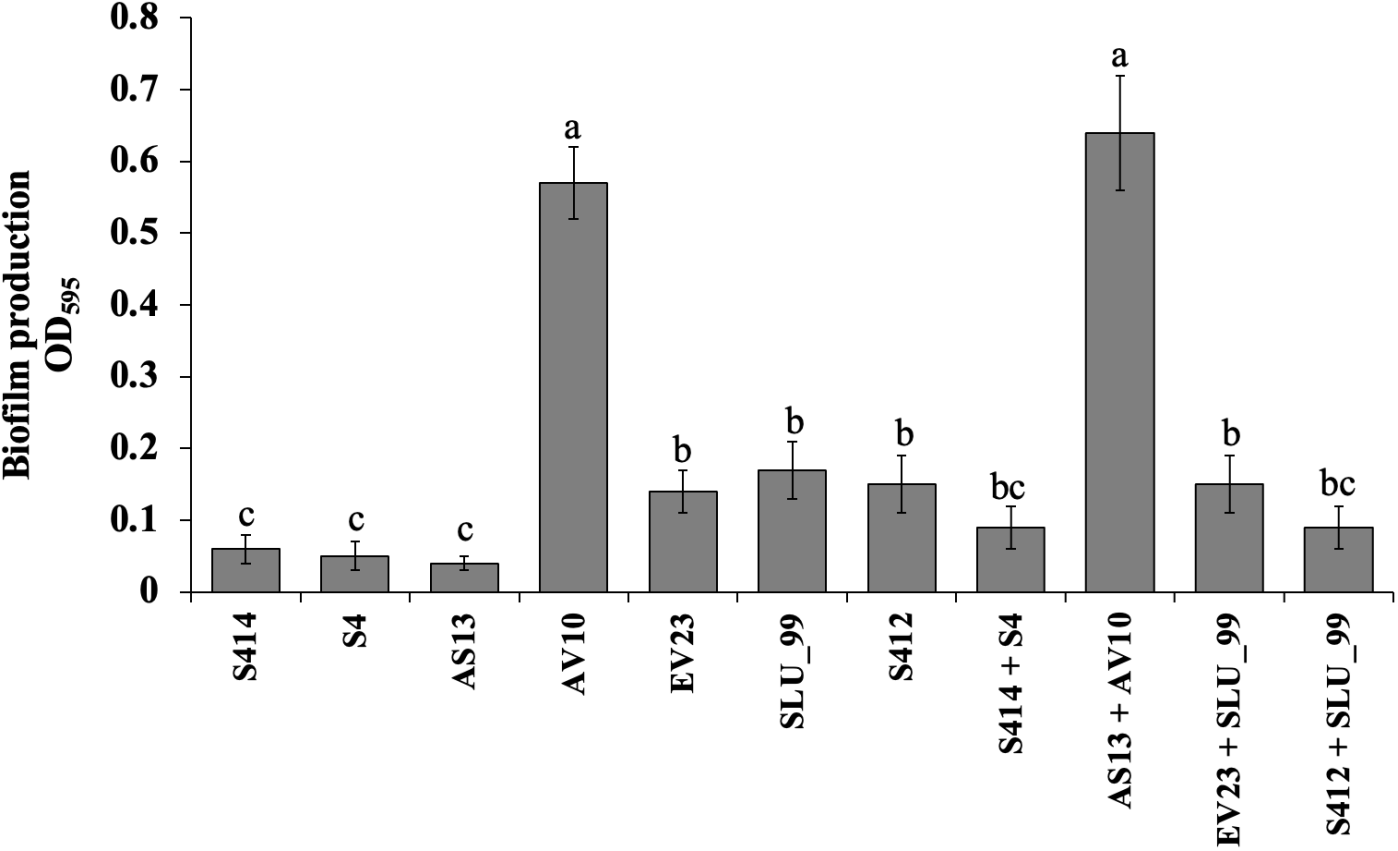
Biofilm production assay of selected bacterial strains using 96-well polystyrene microplates. Data are the means ± standard deviation (SD) of octuplicates per strain. Mean values with different letters indicates a significant difference between strains according to Duncan’s multiple range test at *P* > 0.05.

Various bacteria-mediated biochemical and lytic enzyme production were functionally assayed *in vitro* using specific media. Briefly, the ability of the strain to form a biofilm was examined in 96-well microtiter plates. Although all strains tested were able to form biofilms *in vitro*, there were differences between strains as shown in **Figure 3**. Of the strains tested, AV10 singly and in combination with AS13 exhibited markedly higher biofilm formation in a microtiter plate with an optical density at 597_nm_ of 0.57 and 0.64, respectively (**Figure 3**).

**Figure 4:**
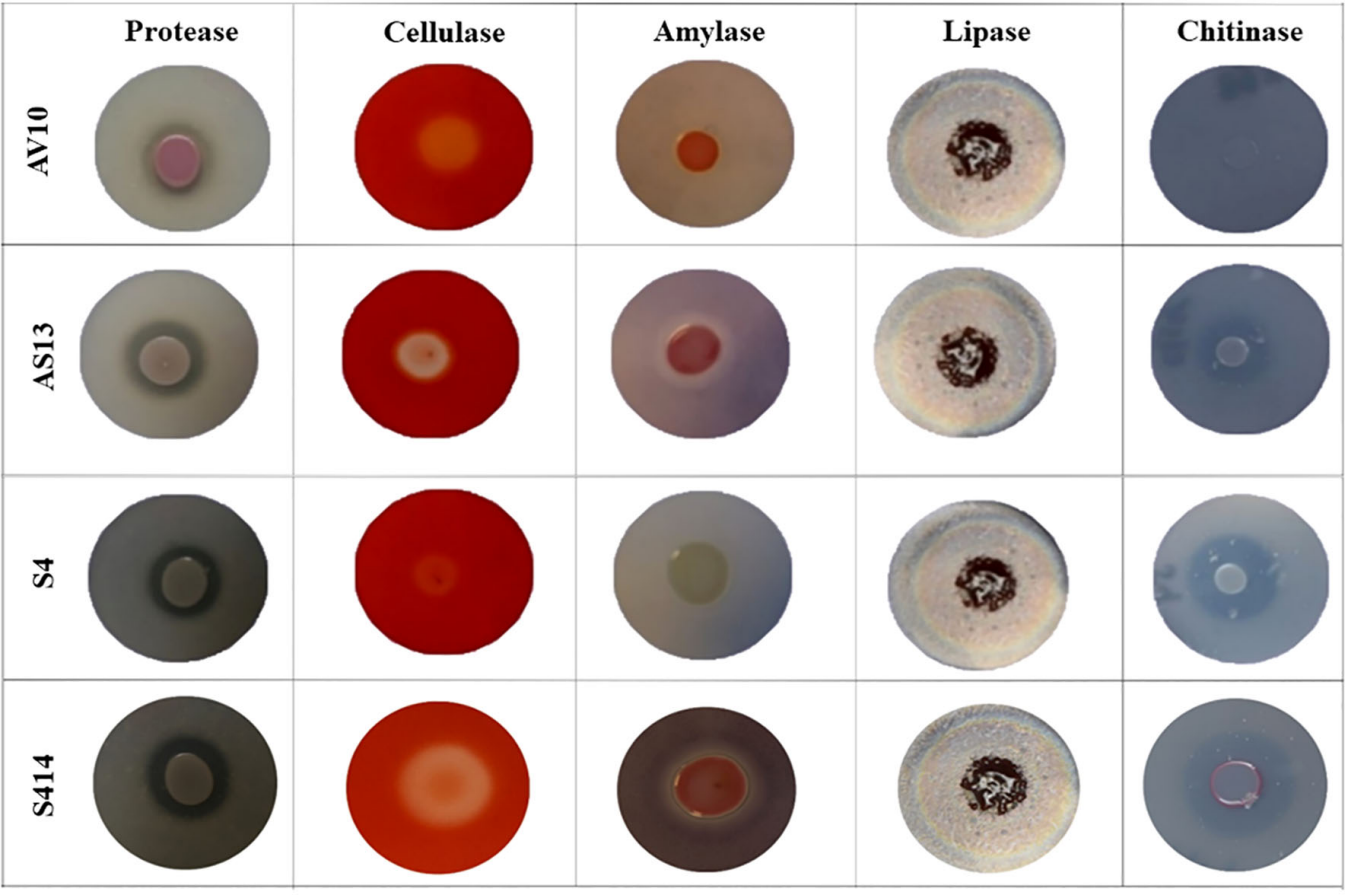
Image showing bacterial production of different lytic enzymes in vitro. Enzyme activity can be seen as zones of clearing or colour change surrounding the bacterial colonies. Strains AV10 and S4 exhibited no amylase activity, and AV10 showed no chitinase activity.

The ability of either single or combined strains to produce lytic enzymes was assessed *in vitro*. Our results reveal that all tested strains were able to produce extracellular protease, lipase, and cellulase (**Figure 4** and **Table 1**). However, there were variations between the strains tested in the formation of distinct halo zones on CMC plates, and in particular the halo zone formed by strain S4 was very weak. In contrast, no clear and distinct lytic halo zone was observed around the AV10 colony that was grown with colloidal chitin and TSA plates supplemented with soluble starch, indicating that this strain was unable to secrete chitinase and amylase enzymes (**Figure 4**). Although the production of hydrogen cyanide was investigated for all selected strains *in vitro*, none of the strains were able to produce it (**Table 2**).

**Table 2:** *In vitro* production of various lytic enzymes by candidate biocontrol bacteria.

Bacterial strains	Protease	Cellulase	Amylase	Lipase	Chitinase	Hydrogen cyanide
S414	+	+	+	+	+	–
S4	+	+	–	+	+	–
AS13	+	+	+	+	+	–
AV10	+	+	–	+	–	–
EV23	+	–	–	+	–	–
SLU_99	+	–	–	+	–	–
S412	+	+	+	+	+	–
S414 + S4	+	+	+	+	+	–
AS13 + AV10	+	+	+	+	+	–
EV23 + SLU_99	+	–	–	+	–	–
S412 + SLU_99	+	+	+	+	+	–

+ Positive result (strains were able to produce lytic enzymes); - negative result (strains were unable to produce these enzymes).

### 3.3 Production of plant growth promoting traits

Selected single and combined strains were examined *in vitro* for traits that are involved in plant growth-promoting activities of bacteria such as IAA, siderophore and ammonia production, and phosphorus solubilisation.

### 3.4 Indole-acetic acid production

Bacterial production of IAA by all strains tested was detected by both qualitative and quantitative assays. The qualitative assay confirmed the production of IAA by the color change of the supernatant from yellow to pink after the addition of the Salkowski reagent. IAA was also quantified in all strains tested in LB broth supplemented with L-tryptophan, and IAA concentration was measured at 530_nm_ using a spectrophotometer (Thermo Fisher Scientific). The result indicated that all strains tested were able to produce IAA in the presence of L-tryptophan (**Table 3**). Strains S4, EV23, and EV23 + SLU_99 produced more IAA, with an average of 0.06 ± 0.001 ug ml^-1^, followed by S412, AS13 + AV10, and S414 + S4 with an average of 0.05 ± 0.002 ug ml^-1^ in media enriched with L-tryptophan.

**Table 3:** Production of different plant growth-promoting traits by bacteria grown *in vitro*.

Bacterial strains	IAA production ug ml^-1^	Siderophore units	PSI	Ammonia production
S414	0.04 ± 0.001^c^	93.51 ± 0.78^abc^	3.21 ± 0.19^bc^	–
S4	0.06 ± 0.001^a^	92.76 ± 0.28 ^cd^	2.79 ± 0.48^c^	+
AS13	0.04 ± 0.001^c^	94.08 ± 0.46^a^	3.44 ± 0.24^bc^	–
AV10	0.04 ± 0.000^c^	92.52 ± 0.55^d^	3.81 ± 0.28^ab^	+
EV23	0.06 ± 0.001^a^	92.87 ± 0.97^cd^	3.28 ± 0.43^bc^	–
SLU_99	0.04 ± 0.001^c^	93.28 ± 0.70^abcd^	4.02 ± 0.41^ab^	–
S412	0.05 ± 0.002^c^	93.85 ± 0.51^ab^	3.45 ± 0.21^abc^	–
S414 + S4	0.05 ± 0.000^b^	93.29 ± 0.38^abcd^	3.22 ± 0.08^bc^	+
AS13 + AV10	0.05 ± 0.000^b^	92.49 ± 0.42^d^	4.25 ± 0.25^a^	+
EV23 + SLU_99	0.06 ± 0.000^a^	93.08 ± 0.62^bcd^	3.97 ± 0.13^ab^	–
S412 + SLU_99	0.04 ± 0.000^c^	93.36 ± 0.42^abcd^	3.67 ± 0.41^ab^	–

+ Represents a positive result; - indicate a negative result. IAA, siderophore units, and PSI have represented means ± standard deviation. Different letters in each column indicate a significant difference between the tested strains according to Duncan’s multiple ranges at *P ≤ 0.05*.

#### 3.4.1 Production of siderophores

The ability of selected strains to produce siderophores was evaluated on chromazurol S (CAS) agar medium. The qualitative test confirmed the production of siderophores by the bacterial strains by the formation of an orange halo zone in the Chromazurol S (CAS) agar. The formation of the halo zone in Chromazurol S (CAS) agar is due to the production of siderophores that remove iron from the dye complex, changing the color of the medium from blue to orange (Devi et al., 2016). The siderophores synthesized by the strains were also confirmed by a quantitative CAS assay, where absorbance was measured at 630_nm_. Further analysis indicated that all tested strains were able to synthesize siderophores in the range of 92.49−94.08% siderophore units (**Table 3**).

#### 3.4.2 Qualitative ammonia production

Ammonia formation by bacterial strains is another important feature associated with plant growth promotion. The qualitative assay result shows that strain S4, AV10 and their combination (S4 + S414, and AS13+AV10) were able to produce ammonia (**Table 3**), as evidenced by the change in color of the inoculated peptone broth to brown after the addition of Nessel’s reagent.

#### 3.4.3 Phosphate solubilization

The ability of selected strains to dissolve Ca_3_(PO_4_)_2_ was investigated using NBRIP medium. A clear and distinct halo zone formed around the colony of the tested strains, indicating the secretion of organic acids into the surrounding medium that dissolves Ca_3_(PO_4_)_2_. In the present study, all strains tested were able to dissolve phosphate in NBRIP agar medium and their phosphate solubility index (PSI) was calculated (**Table 3**).

### 3.5 Biocontrol efficacy of selected strains

**Figure 5:**
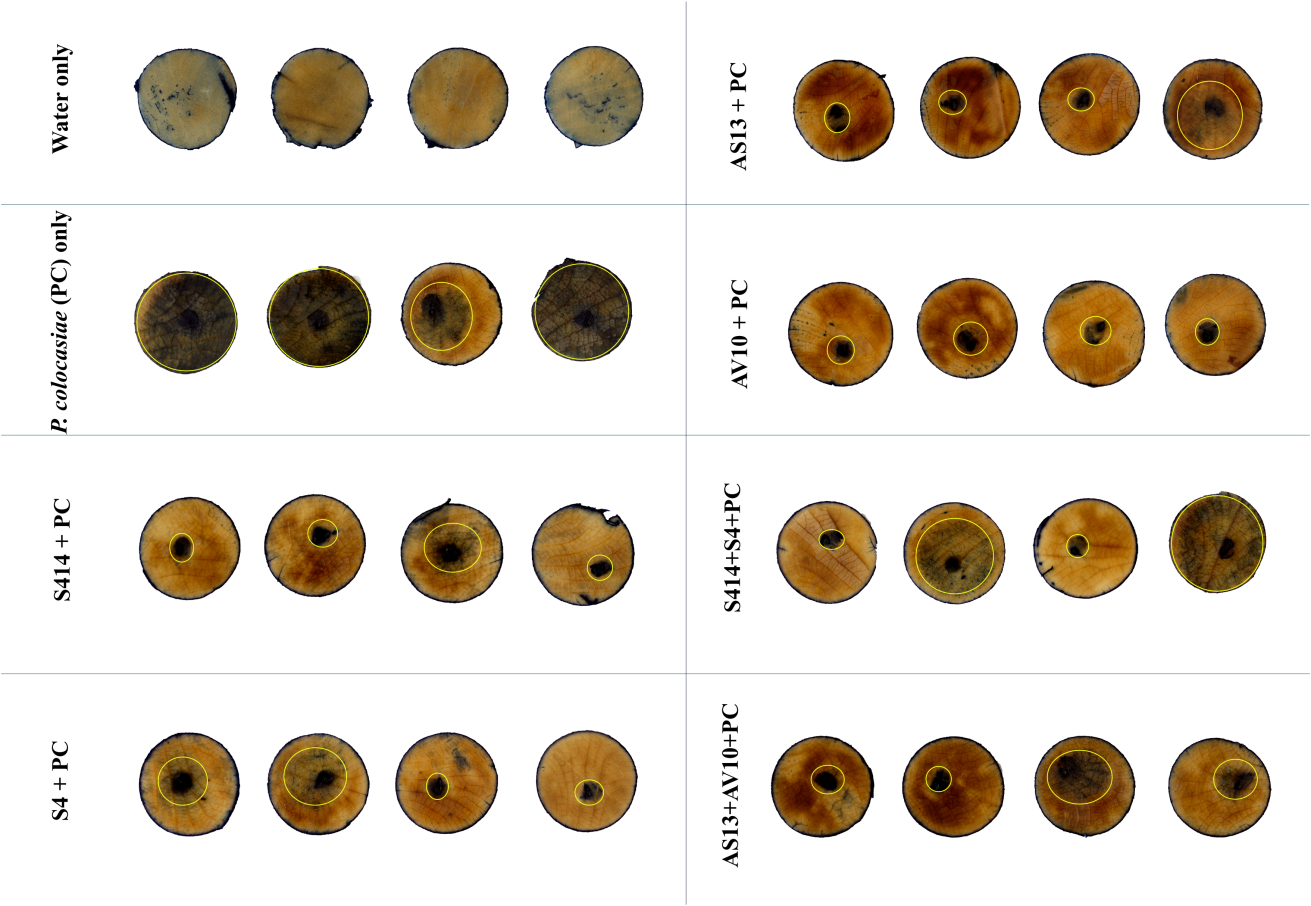
Trypan blue staining of detached taro leaves showing the biocontrol efficacy of selected bacterial strains against *Phytophthora colocasiae*. Taro leaf tissue uninfected by *P. colocasiae* showed no staining, or isolated speckles. The positive control for *P. colocasiae* infection showed strong trypan blue staining that extended beyond the inoculation site. Treatment with biocontrol bacteria limited infection by *P. colocasiae*, evidenced by no trypan blue staining beyond the initial inoculation site. Yellow circles indicate the limit of *P. colocasiae* disease lesions.

**Figure 6:**
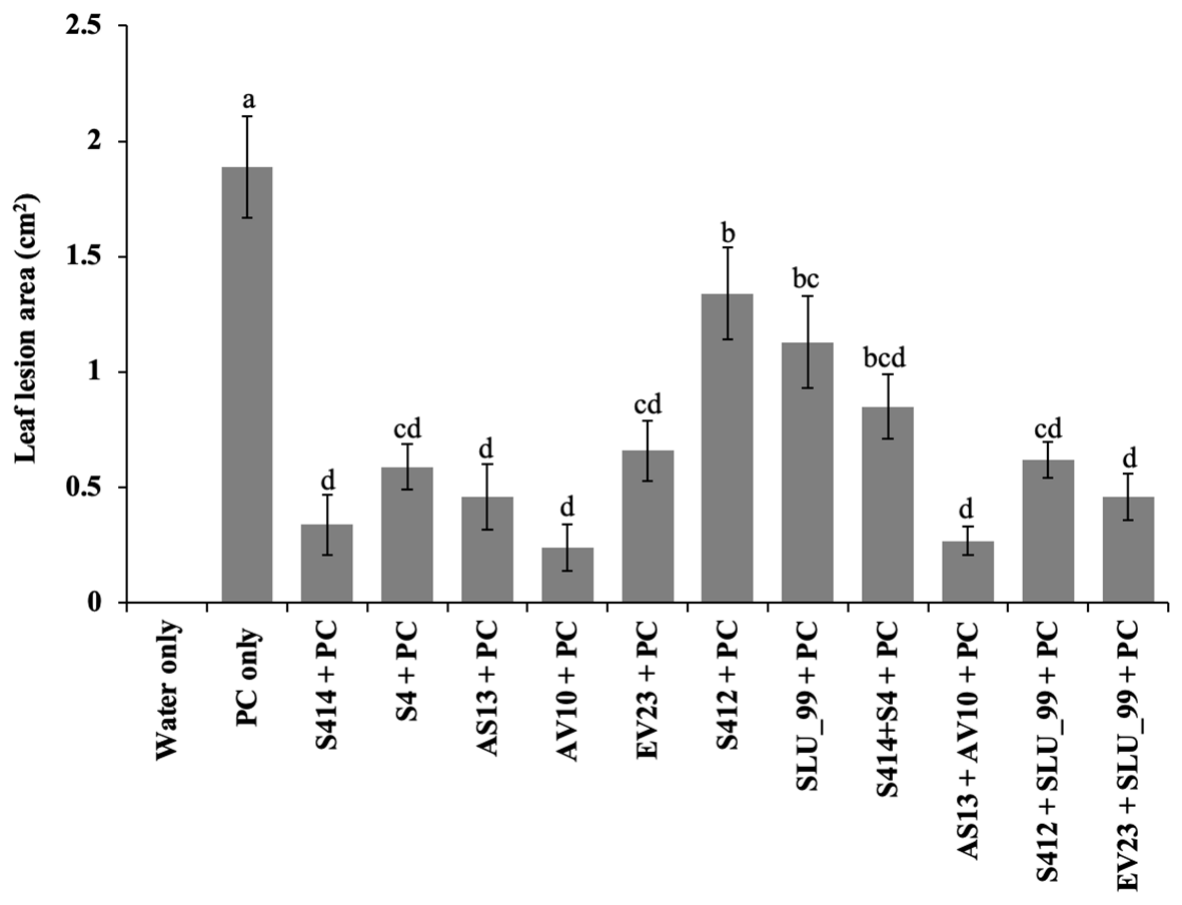
Biocontrol efficacy of bacterial strains on detached taro leaves against *P. colocasiae*. Data are the means ± standard deviation. Means with the same letters show a non-significant difference between the treatments according to Duncan’s multiple ranges at *P ≤ 0.05*.

**Figure 7:**
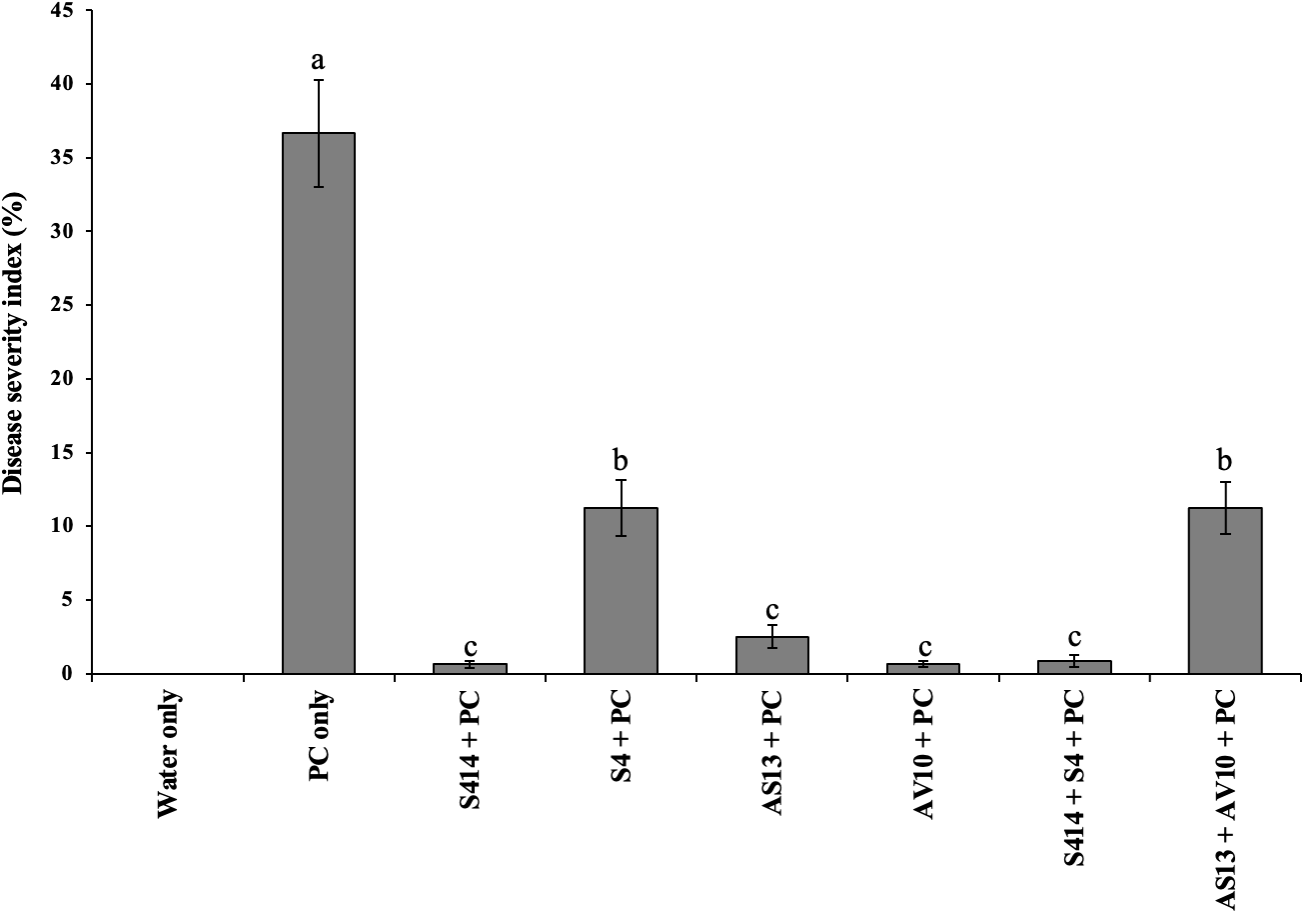
Biocontrol efficacy of bacterial strains against *P. colocasiae* disease development on taro plants under greenhouse conditions. Data are the means ± standard deviation. Means with different letters indicate a significant difference between the treatments according to Duncan’s multiple ranges at *P ≤ 0.05*.

The biocontrol efficacy of selected strains against taro leaf blight disease was further evaluated in DLAs and greenhouse trials. The biocontrol efficacy of tested strains against *P. colocasiae* in DLAs is shown in **Figures 5**, **6**. Taro leaves treated with single or combined strains as consortia developed significantly smaller necrotic lesions compared to the untreated control when *P. colocasiae* was applied alone. However, there were differences between the strains tested in preventing the development of necrotic lesions on taro leaves. Of the strains tested, AS13, AV10, S4, S414, AS13 + AV10, and S414 + S4 displayed more inhibition of necrotic lesions caused by *P. colocasiae* in DLAs. The efficacy of these strains was further studied under greenhouse conditions on whole plants. Similar results were obtained in the suppression of taro blight disease (**Figure 7**). Bacteria-treated taro leaves had a significantly lower disease severity index than untreated controls, which were most affected by the pathogen with disease severity index of 36%. Although the combination of AS13 and AV10 as a consortium showed a similar effect to the single strains in DLAs (**Figure 6**), the effect of the consortium differed from the single strain treatment under greenhouse conditions (**Figure 6**). Under greenhouse conditions, application of S414+S4 as a consortium suppressed TLB disease significantly more than AS13+AV10 at 5 days after treatment, although the effect was reversed in DLAs (**Figure 6**).

### 3.6 Plant growth-promoting efficacy of bacterial strains

The plant growth-promoting ability of either single or combined strains on taro plants was evaluated *in vivo*, and the results are shown in **Table 4**. Analysis of results revealed that inoculation with bacterial strains had a positive effect on taro growth parameters. Application of strain AV10 led to a significant increase in taro plant height (53 cm), compared to untreated control plants (41 cm). Increases in fresh weight, dry weight, root weight, and corm weight of taro plants were observed after treatment with strain AS13 compared to untreated control plants (**Table 4**). Similarly, strain S414 resulted in a significant increase in chlorophyll content. The combination of strains AS13 + AV10 enhanced fresh weight of the plant (**Table 4**). However, the effects of the strain consortium on taro growth parameters are marginal compared to the effects of the individual strains.

**Table 4:** Effect of bacterial strain inoculation on growth parameters of taro plants 30 days post-treatment under greenhouse conditions.

Treatments	Height (cm)	Fresh weight (g)	Dry weight (g)		Root weight (g)	Chlorophyll (µmol m^-2^)	Corm weight (g)
Control	41.14 ± 2.11^b^	48.29 ± 4.44^b^		4.57 ± 0.97^b^	7.71 ± 2.43^b^	43.83 ± 3.67^c^	3.71 ± 3.49^b^
S414	44.71 ± 4.55^b^	52.90 ± 5.63^ab^		7.00 ± 3.26^ab^	8.11 ± 1.83^b^	62.96 ± 9.05^a^	4.86 ± 2.8^b^
S4	45.42 ± 4.35^b^	55.51 ± 5.39^ab^		7.71 ± 2.49^ab^	11.00 ± 4.39^ab^	46.95 ± 3.93^c^	9.43 ± 3.42^ab^
AS13	47.57 ± 3.86^ab^	63.70 ± 8.48^a^		8.86 ± 2.9^a^	15.57 ± 4.99^a^	60.48 ± 5.49^ab^	12.71 ± 4.44^a^
AV10	53.43 ± 5.85^a^	62.43 ± 8.83^a^		7.85 ± 2.79^ab^	11.00 ± 2.11^ab^	49.89 ± 10.58^bc^	5.71 ± 3.15^b^
S414+ S4	46.20 ± 4.49^b^	60.46 ± 6.13^ab^		7.43 ± 1.72^ab^	12.86 ± 2.12^ab^	53.17 ± 6.49^abc^	5.43 ± 4.07^b^
AS13+AV10	45.97 ± 2.94^b^	62.67 ± 7.33^a^		7.71 ± 2.14^ab^	12.71 ± 3.73^ab^	53.49 ± 5.89^abc^	6.00 ± 2.76^b^

Plant height, fresh weight, dry weight, root weight, chlorophyll content, and corm weight are represented as the means ± standard deviation. The same letters in each column reveal a non-significance difference between the treatments according to Duncan’s multiple ranges at *P ≤ 0.05*.

## 4 Discussion

The use of PGPRs as biocontrol agents show great promise for rapid adoption to control plant diseases, including *P. colocasiae*, the causal agent of TLB, as concerns grow about the overuse of agrochemicals in agriculture (Wu et al., 2021; Özdoğan et al., 2022). Biocontrol activities of rhizobacterial strains of the genus *Bacillus* and *Pseudomonas* have been extensively studied against a broad spectrum of plant pathogens (Borriss, 2011; Berger et al., 2015; Dimkic et al., 2022; Ghadamgahi et al., 2022). Currently, research on biocontrol of plant pathogens is being extended to other rhizobacterial genera such as *Serratia*, as these strains have proven to be effective biocontrol agents against a number of plant diseases (Liu et al., 2010; Purkayastha et al., 2018; Kshetri et al., 2019). In the present study, we investigated the biocontrol efficacy of seven bacterial strains (six *Serratia* strains and one *Pseudomanas* strain) and their sixteen dual combinations against *P. colocasiae in vitro*. Our results showed that all tested bacterial strains inhibited the mycelial growth of *P. colocasiae*. The zones of inhibition produced by the tested strains varied, and better reduction of mycelial growth and formation of aerial hyphae structures was observed on detached leaves treated with single and combined strains.

To further evaluate the antagonistic activities of the strains, we tested their cell-free filtrate *in vitro* against *P. colocasiae*. The result showed that the cell-free filtrate inhibited mycelial growth and aerial hyphal structures, indicating the possible activity of secondary metabolites in their cell filtrate. Studies have shown that *Serratia* strains produce different secondary metabolites that can inhibit oomycetes and fungal pathogens by altering their metabolic activity (Su et al., 2016; Clements et al., 2019). On the other hand, *Pseudomonas fluorescens* strains have been reported to suppress *P. colocasiae* by synthesising the secondary metabolite phenazine (Ntyam Mendo et al., 2018). In DLAs, despite ideal conditions (temperature, 22°C, and RH, 72%) favoring *P. colocasiae* infection and growth, the single and combination strains tested reduced necrotic leaf lesions equally, indicating that the combined strains had no additional benefit in reducing *P. colocasiae* disease.

In greenhouse trials, the strains tested suppressed pathogen severity to varying degrees while also improving some aspects of plant growth. Better suppression of disease severity was observed on leaves treated with the single strains AV10, S414, AS13, and combined strains S414 + S4. The presence of these strains prevented pathogen infection from spreading beyond the initial site of inoculation, implying that they may confer host resistance to systemic infection. Our study is in agreement with the findings of Sriram and Misra (2007), who tested different PGPR strains against TLB and reported the reduction in the incidence and severity of the pathogen under polyhouse and field conditions. However, in their study, the rhizobacterial strains tested against TLB were not identified to genus or species level. Numerous authors have also investigated the biocontrol potential of various strains of *Serratia* isolated from rhizospheres in controlling plant diseases caused by other *Phytophthora* spp., and fungal pathogens in tomato (Abreo et al., 2021), potato (El Khaldi et al., 2015), cucumber (Kamensky et al., 2003), pepper (Shen et al., 2007), oilseed rape (Kalbe et al., 1996), and tea (Purkayastha et al., 2018). Moreover, De Vleesschauwer et al. (2009) tested *Serratia plymuthica*, IC1270 against hemibiotrophic and necrotrophic leaf pathogens in rice and observed induced systemic resistance to these pathogens.

In both DLAs and greenhouse trials, the tested strains produce strong and consistent suppression of *P. colocasiae* infection compared to the untreated controls. The combined strains, however, did not suppress *P. colocasiae* disease any better than their individual strains. This could be due to the competition between the strains, or because the strain tested in combination may have variation in disease suppression when used in combination. However, mixing different strains with different mechanisms of action may aid plants deal better with different phytopathogens and abiotic stress conditions. Previously, studies comparing the antagonistic effect of bacterial strain combinations with that of the respective single strain application yielded diverging results. De Vrieze et al. (2018), tested the biocontrol effect of combined *Pseudomonas* strains together with their respective single strains against *P. infestans* on detached potato leaves and observed good efficacy of the combined strains over the single strains. In contrast, Pertot et al. (2017) observed lower efficacy of combined strains compared to their single constituents when using three commercially available biocontrol agents (two fungal-based and one *Bacillus*-based) against *Botrytis cinerea* on grapevines in the field.

In this study, the strains tested were able to produce different lytic enzymes, including protease, cellulase, amylase, lipase, and chitinase, which may be factors in their antagonistic activity against *P. colocasiae*. These enzymes have been reported to be involved in inhibition of cell wall formation, destruction of nucleic acids, inhibition of carbohydrate and protein synthesis, blockage of important metabolic pathways, and induction of systematic resistance in plants (Pang et al., 2009; Etebu and Arikekpar, 2016). According to Gutiérrez-Román et al. (2014), strains of the genus *Serratia* are known to produce the enzyme chitinase, which is known to attenuate fungal infections in plants by degrading the chitin of fungal cell walls. In addition, these bacterial strains have been reported to secrete other lytic enzymes such as protease, cellulase, amylase, and lipase (Van Houdt et al., 2007; Purkayastha et al., 2018), which may be involved in the biocontrol activity of the strains along with chitinases (Richter et al., 2011; Schuck et al., 2014; Jupatanakul et al., 2020). However, in this study, strain AV10 was unable to produce the enzyme chitinase but showed a greater reduction in pathogen growth, which either may be due to the strong biofilm formation of this strain or cellulase that the strain produces that may target the cellulose cell wall of *P. colocasiae*.

Numerous studies have shown that PGPR, including *Serratia* strains, colonize the root tips of plants and form a biofilm-like structure containing predominantly carbohydrates, proteins, and extracellular DNA that serves as a protective layer against various stresses, including plant diseases (Rice et al., 2005; Liu et al., 2011; Haque et al., 2020; Speranza et al., 2020). Inoculation of wheat with biofilm-forming PGPR strains improved plant growth even under stressful conditions (Amna et al., 2019). In contrast, none of the strains tested in this study was capable of producing hydrogen cyanide (HCN). The inability of strains to produce HCN is considered a desirable feature of PGPR because HCN inhibits plant growth and development by acting as a potential inhibitor of enzymes involved in cytochrome oxidation and can also block photosynthetic electron transport by binding to the protein plastocyanin (Bakker and Schippers, 1987; Kremer and Souissi, 2001; Purkayastha et al., 2018).

The tested strains not only inhibited the symptoms of the pathogen, but also improved some vegetative growth parameters of the taro plant. The improvement in plant growth parameters could be due to the strains possessing growth promoting properties such as IAA, siderophores and phosphorus solubilizing potential. In our study, all strains tested were able to produce IAA *via* L-tryptophan-dependent metabolic pathways. However, the production of this phytohormone did not directly correlate with plant growth, as some combined strains (AS13 + AV10) produced a higher amount of IAA but resulted in lower taro plant growth than strain AS13 alone, suggesting that bacterial-mediated production of IAA alone does not necessarily lead to growth promotion. PGPR, including *Serratia* strains, have been reported to produce IAA in both L-tryptophan-dependent and independent pathways that coordinate various developmental processes in plants such as cell division, seed germination, photosynthesis, root growth, and also protect plants from various stresses (Spaepen and Vanderleyden, 2011; Özdoğan et al., 2022). Similarly, all strains tested in this study were able to synthesise siderophores, which may increase the competitive advantage of the strains in colonising their host roots and inhibiting plant pathogens by sequestering essential iron in an iron-deficient environment (Yasmin et al., 2016; Caulier et al., 2018; Sun et al., 2022).

Moreover, all strains tested efficiently dissolved tri-calcium phosphate in NBRIP medium *in vitro*, suggesting that these strains are more likely dissolving phosphorus salts, some of which the strains consume themselves and the rest of which is passively available to plant roots. PGPR strains have been reported to be involved in the natural phosphorus cycle by dissolving different types of phosphorus in different soils in a pH-dependent manner (Adnan et al., 2017; Alori et al., 2017), making them available to plants (Oteino et al., 2015). In this study, strains AV10, S4, AS13 + AV10, and S414 + S4 were able to produce ammonia. The production of ammonia by PGPR strains is another important feature to meet the nitrogen demand of host plants (Marques et al., 2010; Bhattacharyya et al., 2020). Ammonia produced by beneficial microbes also serves as a defense against harmful microorganisms by directly inhibiting their colonization of host plants and further limiting the germination of fungal spores (Yadav et al., 2010). Overall, the strains tested produced strong disease suppression and improved vegetative growth, but mixing some of these strains did not provide additional benefits in disease suppression and plant growth improvement when compared to individual strains.

## 5 Conclusion

Many PGPR strains have been isolated and tested for their antagonistic activity against various plant pathogens, but very little information is available on biocontrol of *P. colocasiae* with PGPR strains. In summary, our study clearly shows that the tested strains have strong biocontrol activity against TLB disease and improve plant growth. Hence, single strains (viz., S414, S4, AS13, and AV10) and AS13 + AV10, S414 + S4 could be used as a new biocontrol strategy for integration into a sustainable TLB disease control program. However, *in vitro* and *in vivo* antagonist tests do not always correlate with biocontrol efficacy of bacterial strains under field conditions. Therefore, further studies should be conducted to investigate the biocontrol efficacy and plant growth promoting effects of these promising strains before integrating them into a sustainable TLB disease control program.

## Data availability statement

The original contributions presented in the study are included in the article/**Supplementary Material**. Further inquiries can be directed to the corresponding author.

## Author contributions

Conceptualization and designing the experiment: RV, FG and SW. Methodology: RV, RB, FG, BK. Data validation and analysis: RV, RB, FG, BK. Investigation: BK and FG. Resources: RV, RO, RB and PK. writing - original draft preparation: BK. Writing - review and editing: RV, SW, RB, RO, PK and BK. Supervision and project administration: RV, RB, SW, RO, PK. Funding acquisition: RV, RB, PK, and RO. All authors contributed to the article and approved the submitted version.

## Funding

This research work was supported by the Swedish Research council (2019-04270) and SLU Partneskap Alnarp.

## Acknowledgments

SCW acknowledges funding from the Scottish Government Rural and Environment Science and Analytical Services (RESAS) Division. IITA acknowledges funding for plant health research from the Plant Health Initiative funded by the CGIAR Trust Fund donors.

## Conflict of interest

The authors declare that the research was conducted in the absence of any commercial or financial relationships that could be construed as a potential conflict of interest.

## Publisher’s note

All claims expressed in this article are solely those of the authors and do not necessarily represent those of their affiliated organizations, or those of the publisher, the editors and the reviewers. Any product that may be evaluated in this article, or claim that may be made by its manufacturer, is not guaranteed or endorsed by the publisher.

## Supplementary material

Supplementary Figure 1
*In vitro* inhibitory activity of bacterial cell-free filtrate against *P. colocasiae*. **(A)** Representative image showing inhibition of mycelial growth of *P. colocasiae* in cultures with cell free filtrate. The control culture showed uniform hyphal growth with the formation of numerous aerial hyphae, while the cell filtrate-enriched CMA inhibited radial hyphal growth of the pathogen. **(B)** Antagonistic activities of single or combined cell-free filtrates on *P. colocasiae*. Data are the means ± standard deviation. Means with different letters indicate a significant difference between the treatments according to Duncan’s multiple ranges at *P ≤ 0.05*.
