# Supplementary figures and images for: Antagonistic and plant growth promotion of rhizobacteria against Phytophthora colocasiae in taro
Source: Front Plant Sci. 2022 Dec 2;13:1035549. doi: 10.3389/fpls.2022.1035549 (PMC9755733; doi:10.3389/fpls.2022.1035549)

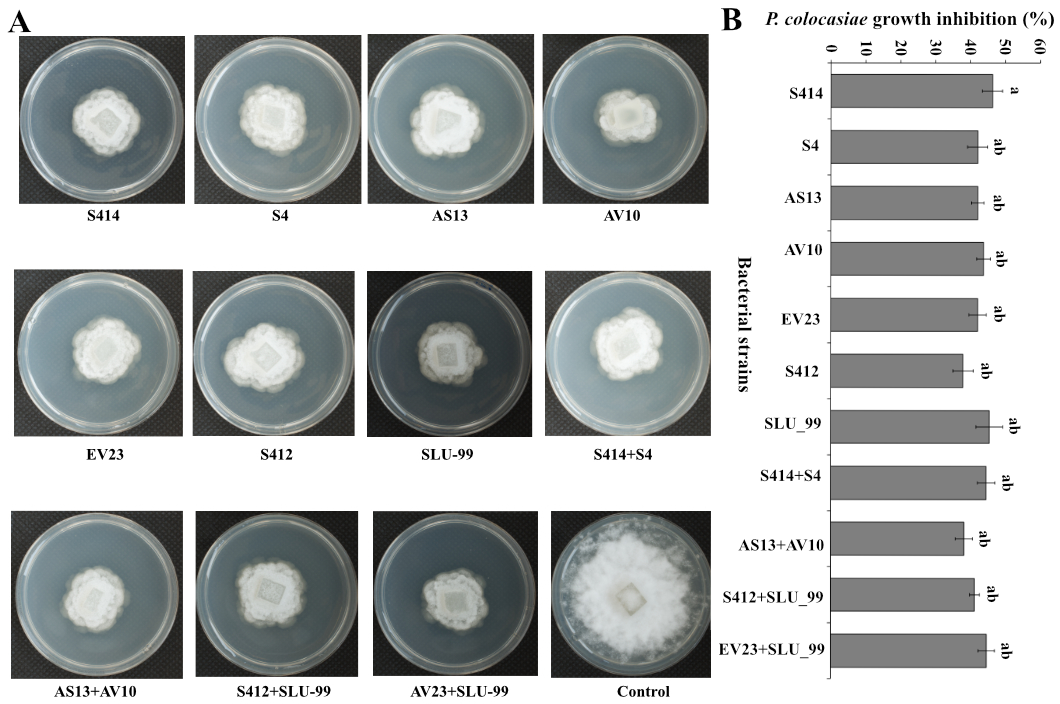

Supplement: Supplementary Figure 1 — In vitro inhibitory activity of bacterial cell-free filtrate against P. colocasiae. (A) Representative image showing inhibition of mycelial growth of P. colocasiae in cultures with cell free filtrate. The control culture showed uniform hyphal growth with the formation of numerous aerial hyphae, while the cell filtrate-enriched CMA inhibited radial hyphal growth of the pathogen. (B) Antagonistic activities of single or combined cell-free filtrates on P. colocasiae. Data are the means ± standard deviation. Means with different letters indicate a significant difference between the treatments according to Duncan’s multiple ranges at P ≤ 0.05. [file Image_1.tiff]
